# In Situ Thermogravimetric Analysis of Curved Surfaces During High-Temperature Oxidation

**DOI:** 10.3390/ma18112463

**Published:** 2025-05-24

**Authors:** Megan Kendall, Michael Auinger, Cadyn L. J. Robinson, Chris Owen, Elizabeth Sackett

**Affiliations:** 1 Department of Materials Science and Engineering, Swansea University, Swansea SA1 8EN, UK; t.robinson.953474@swansea.ac.uk (C.L.J.R.); e.sackett@swansea.ac.uk (E.S.); 2 WMG, University of Warwick, Coventry CV4 7AL, UK; m.auinger@warwick.ac.uk; 3 Tata Steel (Tubes), Corby NN17 5UA, UK

**Keywords:** oxidation, steel, heat treatment, thermogravimetric analysis, modelling, geometric effects

## Abstract

Conveyance tube manufacturing via a hot-finished, welded route is an energy-intensive process that promotes the rapid surface oxidation of curved surfaces. Previous studies have used computational and theoretical techniques to assess the oxidation of curved surfaces. However, experimental techniques for assessing the oxidation of curved surfaces, as well as for validating existing computational and analytical studies, have significant limitations that impact their ability to accurately recreate industrial processes. The challenges of thermogravimetric analysis (TGA) for in situ tests for the oxidation of cylindrical geometries were investigated, using an as-welded conveyance tube, and compared to an equivalent tube normalised in industry as well as computational predictions for the same geometry and thermal conditions. A core element of this work was the use of a refractory dummy sample to quantify thermal buoyancy and flow-induced vibration. There was a strong agreement between the oxide mass gain predicted by a computational model compared to that of the TGA sample, with only a 5% discrepancy. However, oxide thickness gain, measured using electron microscopy, showed poor agreement, particularly when comparing industrial and experimental results. This was attributed to the need for further work to account for transient heating, oxide porosity, atmospheric composition variation, and the effect of thermomechanical operations during conveyance tube manufacturing, e.g., hydraulic descaling.

## 1. Introduction

**Figure 1:**
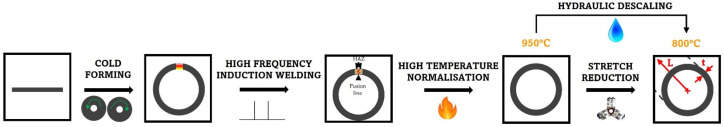
The schematic sequence of the conveyance tube-manufacturing process (figure reproduced from the work by Kendall et al. [7]).

Curved surfaces and geometries are integral to the key shapes used in engineering [1] and are prevalent in several engineering applications involving high-temperature oxidation during manufacturing, installation, and/or service, e.g., HVAC systems and petrochemical transport, where high pressures, e.g., refrigeration, and temperatures, e.g., steam system, are possible [2,3,4,5,6]. The manufacturing process must be designed to achieve the material and mechanical properties necessary for these service conditions. A summary of the manufacturing process is shown in Figure 1, and the full details of which have been discussed by Kendall et al. [7].

Component damage and failure due to surface defects associated with phenomena such as oxidation have consequences, such as losses of up to 3% by weight occur due to scaling alone (equivalent to approximately 500 tonnes of feedstock loss per warehouse batch) [8]. There are also aesthetic implications due to either the compaction of scale on the substrate surface (‘rolled-in effect’) and/or the inadequate adherence of value-added coatings, e.g., anti-corrosion paint, and in-service surface performance problems [9]. The resulting inconsistent scale adhesion not only appears to give a poor surface finish but also may lead to complications for those customers who wish to apply additional paint or coat the tubes. Furthermore, scale removed either by descaling and/or spalling tends to disperse into the manufacturing environment, causing damage and contamination to product and plant alike via oxide particle entrainment and third-body abrasion [10,11,12]. However, it is a challenge for plants to optimise their processes to maximise product quality, capacity, process flexibility, and cost-effectiveness whilst high-temperature oxidation on curved surfaces is still poorly understood. It should be noted that, throughout this paper, ‘2D’ refers to the consideration of the two dimensions (radial, r, and circumferential, θ) of the tube cross-section [3,5,13,14,15] as opposed to 2D oxidation phenomena [16,17,18,19].

Kendall et al. sought to address the issue of oxidation within conveyance tube manufacturing computationally, particularly the additional challenges associated with cylindrical geometries, in their work [7]. They explored the potential for a computational approach for modelling high-temperature oxidation, particularly on curved surfaces. Their parametric study demonstrated the influence of wall thickness on oxidation rate via changes in diffusion path length and how the 2D nature of the model, in isolation, cannot capture the effect of outer diameter changes in surface area. However, this effect has been shown to be limited to tubes with inner radii of less than 200 mm. Beyond this critical value, the scale of diffusion is small enough to render the effect of the radial coordinate in the diffusion negligible, and the solution approaches that of a planar geometry.

Manning [20] theoretically investigated the effect of curvature, specifically on tube surfaces, on scale adherence, highlighting that scale failure not only causes spallation but can also initiates transition between kinetic regimes (parabolic, linear, logarithmic, etc. [21,22,23,24]) during high-temperature oxidation. Curved geometry effects can contribute to both the resulting strain applied on the oxide layer due to curved surface oxidation and changes in scale resistance due to external strain application. In contrast to uniform oxidation on a flat surface where the scale is normally unrestrained and able to accommodate volume expansion, curved geometry is often responsible for stresses in addition to those generated by epitaxial restraint (although the latter was too complex and specific to consider in Manning’s work). Furthermore, for curved surfaces, even with an unconstrained free surface, the introduction of new oxide between the metal and existing oxide leads to stresses and strains at the surface radii as the metal retreats via consumption and loses contact with the existing oxide. In other words, any surface curvature (where the minimum ratio between the grain size and surface curvature is 1:20 [25]) inhibits the oxide from following volume changes in the oxidising substrate, leading to elastic and creep deformation [20]. Furthermore, the hoop stress found in planar examples is extended to a 2D state to include radial compressive stress, which Clarke highlights is maximum at the metal–scale interface [25]. However, Stott and Atkinson argue that creep and plastic deformation phenomena are often neglected in quantitative models [26]. Finally, Schutze commented on the effect of intrinsic growth stresses on perceptibly damage-free oxide scale after high-temperature treatment [4].

Kendall et al. highlighted the challenges of experimental investigation of curved surface oxidation, particularly the limitations of equipment used in in situ oxidation investigations [7]. Thermogravimetric (TGA) furnaces, which provide continuous mass gain data, are often limited in their sample size capacity, so only very small tubes can be used, or tubes have to be sectioned, which causes machining-induced residual stress [27,28]. Furthermore, there are issues with TGA measurements as they assess the total oxidation on a sample and neglect the effect of the gas flow on the extent of oxidation in each part of the sample geometry, as shown in the work by Mori et al. [29]. Therefore, any experimental value derived from TGA curves is an average of the entire geometry, which does not address the differences between the surface on which the flow is incident and the surface facing away from the flow. The strength of modelling is its ability to distinguish between the two surfaces, and this is the reason for focusing on modelling studies and then subsequently correlating their results with the total oxidation thickness measured after heat exposure (the latter being subject to errors due to the spallation of poorly adherent oxides). Furnaces with a larger sample size capacity do not usually have TGA equipment so rely on pre- and post-heat treatment high-resolution mass balance measurements of the sample. High-temperature oxides are known to be brittle as observed by the authors and as reported in the literature [30,31,32,33]. This brittle nature makes scale spalling inevitable so that material is lost between transferring the sample from the furnace to a mass balance, in addition to losses during sample cooling before it is removed from the furnace due to thermal mismatch between the metal and oxide. However, all computational (and theoretical) modelling relies on idealised assumptions to obtain a sufficiently accurate output within economic and time constraints and inevitably requires experimental validation to confirm the credibility of its predictions both for and beyond the original context and response [34].

The work in this paper aims to address the need to develop robust experimental methods for investigating the oxidation of curved surfaces, in an attempt to validate the computational model of oxidation kinetics for cylindrical geometries developed by Kendall et al. [7]. The limitations of conventional experimental techniques are identified and strategies are proposed to mitigate any issues. The resulting data are used to assess the viability of their model, as well as assess the capabilities of the experimental techniques discussed.

## 2. Materials and Methods

### 2.1. Materials

The materials under investigation during in situ oxidation tests for validation purposes were the two grades of low-carbon, low-alloy steel P235GH (compliant with the material specification BS EN10217-2) [35]. The specification given in BS EN10217-2 is outlined in Table 1. Nose crop samples of both grades were supplied by Tata Steel Tubes UK Ltd. (Corby, UK).

Samples of 15 nominal bore (n.b.) P235GH conveyance tubes (outer diameter (OD) = 21.9 mm, wall thickness ($r_{wall}$) = 3.0 mm, length (L) = 25 mm) in an as-welded and post-normalised state were supplied by Tata Steel UK Ltd. The possible range of conveyance tube dimensions is wide depending on intended service application [36], but using the 15 n.b. sample allowed for unrestricted movement within the furnace work tube (ID = 90 mm) and was suitable for conventional 30–32 mm diameter mounting moulds and scanning electron microscopy sample holder limits.

### 2.2. Thermogravimetry

Thermogravimetric analysis (TGA) is the measurement of mass change as a function of temperature or time at a constant temperature. The combination of the mass balance and furnace forms the thermobalance system [37]. There is also a trade-off between selecting a sample size that is large enough (≥1 g) to leave residue suitable for further chemical testing but small enough (can be as low as 1 mg) to maximise the potential for a steady and uniform temperature distribution in the sample.

**Figure 2:**
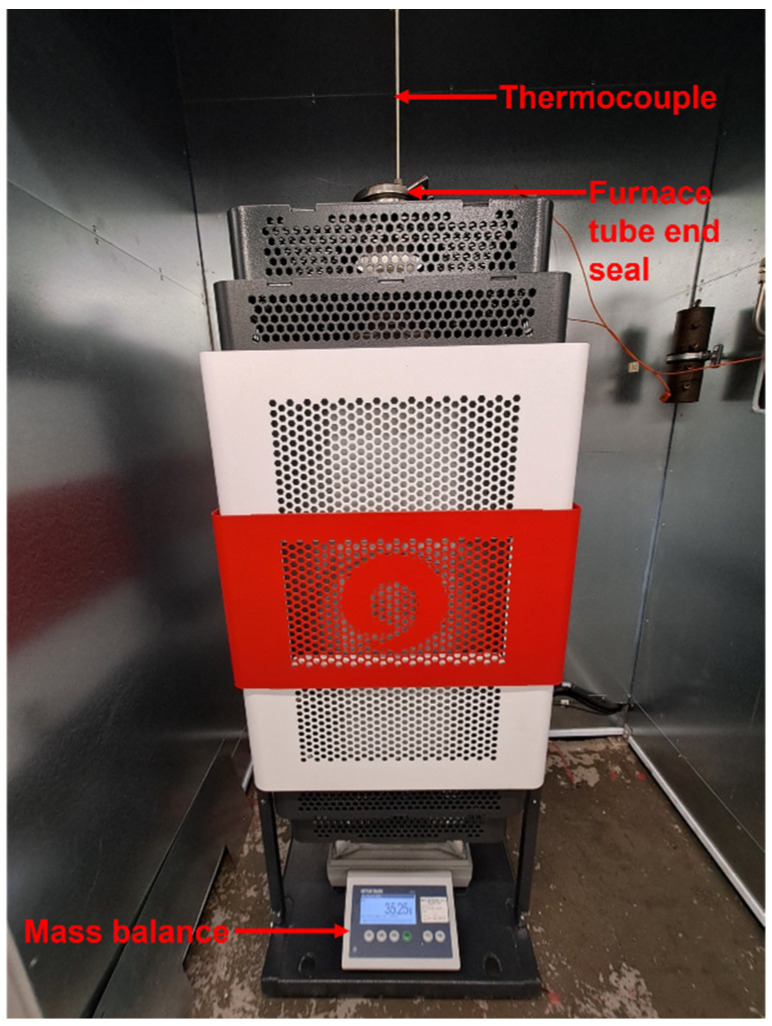
Carbolite Gero Ltd. high-temperature TGA tube furnace used to perform furnace replication experiments and obtain continuous thermogravimetric data.

Mass gain data were obtained from the vertically mounted Carbolite Gero Ltd. (Hope Valley, UK) high-temperature TGA tube furnace (model TF1 16/100/450) in the Simulation and INtegrity Testing in Extreme Conditions (SINTEC) laboratory within the Steels and Metals Institute (SaMI) at Swansea University (see Figure 2), with a heating capacity of up to 1400 ± 5 °C (see Appendix A).

During furnace operation, the mass balance (Mettler Toledo ICS425K-15LA, Leicester, UK) continuously weighs the entire work tube in addition to the sample itself, overall providing continuous mass gain data for the sample. A type ‘R’ (platinum–13% rhodium/platinum) thermocouple is attached to the heating chamber via a ceramic tube to obtain continuous temperature data. An as-welded sample was used during TGA testing. The tube sample was weighed on a Fisher Scientific 0.0001 g precision mass balance (Loughborough, UK) before testing for calibration purposes. The sample used had an initial mass of 34.1996 g (although this level of precision was far greater than that of the furnace mass balance of 0.05 g). The as-welded sample had a single through-thickness hole drilled into its wall to allow its suspension from the ceramic radiation shield arrangement using a nichrome wire. A 15 min period was left between switching the balance on and starting any weighing operations, to ensure the settling of the mass balance signal after the sample mounting. The single thermal cycle comprised a 5 °C·min^−1^ ramp to 1000 °C, followed by a 15 min dwell, and finally a 5 °C·min^−1^ cooling ramp.

Thermally induced gas flow phenomena were proposed to be the greatest proponents of both noise and mass loss in the sample during heating and cooling periods. The large temperature change in the thermal cycle used makes the gas in the furnace environment compressible and therefore demands conservation of momentum (see Equation (1)) for a given displacement, $x_{i}$, and time, t, so that the ratio of velocity, u, in a given direction, i, and temperature is constant (see Equation (2)) [38,39,40]. Density, ρ, varies spatiotemporally whilst pressure, P, varies only spatially. The acceleration due to gravity on Earth ($g_{i}$) = 9.8 m·s^−2^ [41,42]. For a temperature change, $T_{2}-T_{1}$, from ambient ($T_{1}$ = 25 °C) to normalisation ($T_{2}$ = 1000 °C), there is a velocity increase of a factor of 4.3, i.e., $\frac{v_{2}}{v_{1}}=4.3$. Newton’s Second Law [43] indicates that the acceleration of the furnace gas enacts an instantaneous force on the sample and initiates damped free vibration [44].

(1) $$\begin{array}{c} \frac{\partial \rho }{\partial t}+\frac{\partial }{\partial x_{i}}\left(pu_{i}\right)=0\\ \frac{\partial }{\partial t}\left(\rho u_{i}\right)+\frac{\partial }{\partial x_{j}}\left(\rho u_{i}u_{j}\right)+\frac{\partial P}{\partial x_{i}}-\rho g_{i}=0 \end{array}$$

(2) $$\frac{u_{1}}{u_{2}}=\frac{T_{1}}{T_{2}}$$

**Figure 3:**
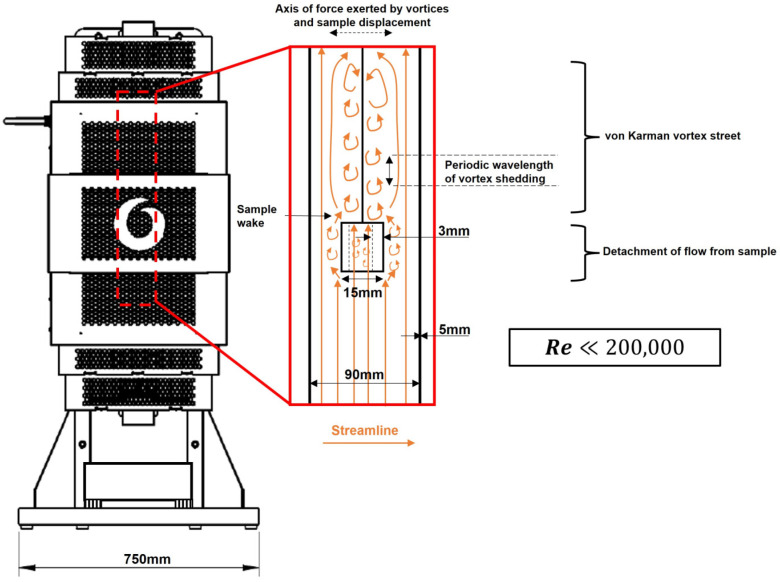
The schematic of the streamline profile induced during vortex-induced vibration (VIV), within the furnace work tube, associated with laminar (Re≪200,000), viscous thermally induced gas flow around the sample blunt body.

During the isothermal period, the fluid around the sample flows at a steady speed well below that needed to reach its critical Reynolds number (Re≪200,000) [45], i.e., flow is laminar, and the walls of the sample act as blunt bodies that retard the flow. The flow becomes detached from the sample in its wake region. Flow detachment from the sample promotes the formation of a von Karman vortex street: a series of counter-rotating vortices that are shed periodically from the sample into its wake (see Figure 3) [46].

The von Karman vortex street exerts a resultant sinusoidal lift force on the sample, perpendicular to the gas flow direction, which can excite the sample into forced vibration if the vortex shedding frequency is close to the sample resonant frequency [45]. This is known as vortex-induced vibration (VIV) [47], and there are specific examples of this vibration occurring around long blunt bodies [48,49].

The overall consequence of these vibration phenomena is damped free vibration due to the thermal buoyancy of the sample in heating and cooling periods, and a pendulum effect from the forced vibration due to VIV in the isothermal period, if the gas excitation frequency is equal to the tube resonant frequency [50]. Therefore, mass balance fluctuations occur due to force balance changes. Although decreasing the gas flow rate could reduce the impact of vibration, the ambient environment means there are limited opportunities to control intake flow velocity, but an alternative solution would be to use a chain instead of a single wire to reduce the effective pendulum length.

Without the ability to control the gas flow, the alternative is to run a dummy test on an identical-geometry high-temperature refractory sample, i.e., a sample that will not gain mass due to oxidation. The instantaneous oxide mass gain, $∆m_{ox,t_{i}}$, at time, $t_{i}$, is therefore obtained by subtracting each instantaneous mass measurement in the dummy test, $m_{alumina,t_{i}}$, arguably due to the gas flow effects discussed, from the original steel tube test, $m_{steel,t_{i}}$ (see Equation (3)).

(3) $$∆m_{ox,t_{i}}=m_{steel,t_{i}}-m_{alumina,t_{i}}$$

**Figure 4:**
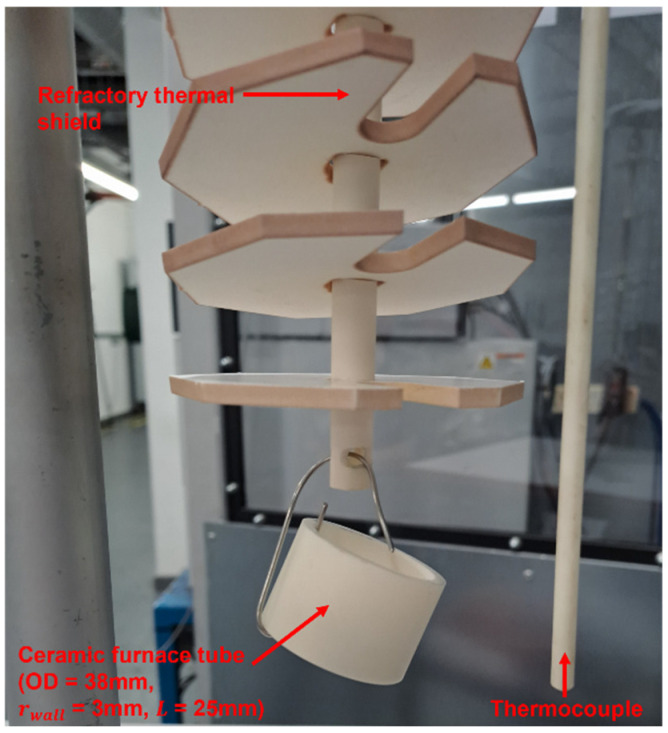
The ceramic furnace tube dummy sample (38 mm outer diameter (OD), 3 mm wall thickness $\left(r_{wall}\right)$, and 25 mm length (L)) suspended from the refractory shield using a nichrome wire.

A dummy sample was run using an alumina (Al_2_O_3_) furnace tube with OD = 38 mm, $r_{wall}$ = 3 mm, and L = 25 mm. Its initial mass, 33.4823 g, was measured using a 4 decimal place (d.p.) Fisher Scientific mass balance. No net mass gain was anticipated during the dummy cycle except for the oxidation of the nichrome suspension wire, a minimum length of which was used to minimise any pendulum effect (see Figure 4). The total mass of the sample and wire was 34.4524 g. It should be noted that, in order to comprehensively explore the interaction of tube geometry and gas flow dynamics, and due to the brittle nature of the ceramic tube material, the nichrome suspension wire was attached through the bore and around the entire tube thickness, but it was not secured to allow it to be removed post-treatment, instead of drilling a suspension hole as in the case of the steel sample.

**Figure 5:**
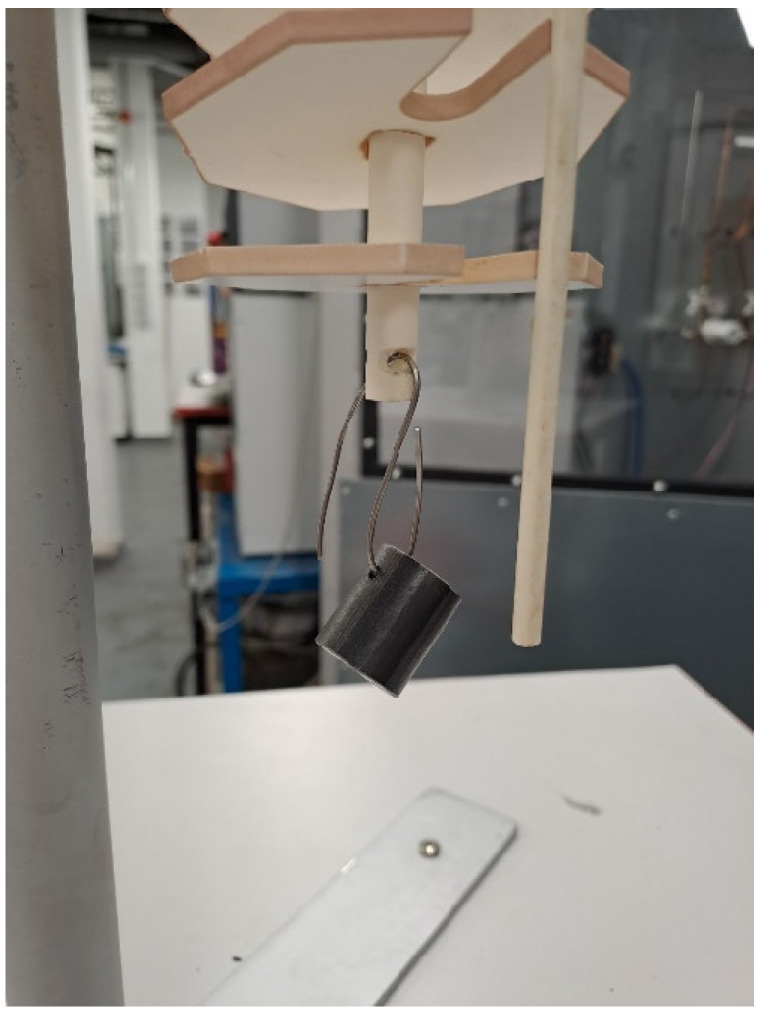
Pre-test setup of 15 nominal bore (n.b.) sample.

When testing the 15 n.b. sample, the suspension wire was attached via a drilled through-thickness hole in the wall, although this meant accepting a greater axial tilt in the sample during testing (see Figure 5). Not only is position control for the sample important from a gas flow perspective, but also is ensuring that the thermocouple position achieves the most accurate sample temperature value as the spatial temperature profile in the furnace work tube is unlikely to be uniform [14,51].

Matching the dummy and real sample masses is more important than matching the geometry when considering the case of thermally induced damped free vibration as mass, m, is a key variable (alongside system stiffness, k) in determining the damping ratio, ζ, and natural frequency, $\omega_{0}$, (see Equations (4) and (5)) of the governing damped harmonic oscillator motion equation for sample displacement, x, as a function of time, t (see Equation (6)) [44]. The forcing function, F, a constant energy source associated with VIV, although also influenced by mass via natural frequency, is more influenced by the energy source of the continuous convective gas flow and its interaction with sample geometry.

(4) $$\zeta =\frac{c}{2\sqrt{mk}}$$

(5) $$\omega_{0}=\sqrt{\frac{k}{m}}$$

(6) $$\frac{d^{2}x}{dt^{2}}+2\zeta \omega_{0}\frac{dx}{dt}+\omega_{0}^{2}x=F$$

The discrete Fourier transform (DFT) (see Equation (7)) can be used to express periodic time-series data, x(t), as a frequency spectrum, X(k), where k is the product of time, t, and frequency, ω [52]. Given that periodic functions of time can be expressed as the sum of a number, N, of discrete sine function components with their own amplitude, $A_{n=1\ldots N}$, frequency, and phase angle, ϕ (see Equation (8)), the forcing frequency, and any associated harmonics, can be identified from equivalent frequency domain data [53].

(7) $$X(k)=\sum_{n=0}^{N-1}\;x(t)e^{-i\frac{2\pi kn}{N}}⟺x(t)=\frac{1}{N}\sum_{k=0}^{N-1}\;X(k)e^{j\frac{2\pi }{N}nk}$$

(8) $$x(t)=A_{0}+A_{1}\sin\left(\omega t+\phi_{1}\right)+A_{2}\sin\left(2\omega t+\phi_{2}\right)+A_{3}\sin\left(3\omega t+\phi_{3}\right)+\cdots ,$$

The fast Fourier transform (FFT) in-built algorithm in MATLAB R2024b was used to evaluate the frequency domain data using the noisy isothermal region from the original TGA time-series data. TGA data quality was assessed using the signal-to-noise ratio, SNR, and coefficient of variance, CV, values based on the calculations of mean, μ, and standard deviation, σ (see Equation (9)).

(9) $$SNR=\frac{\mu }{\sigma };CV=\frac{100}{SNR}$$

Samples were subjected to a representative heating cycle that was representative of the conveyance tube normalisation treatment, i.e., 1000 °C for 15 min. The selected heating rate was set at 5 K·min^−1^ (the maximum rate available within the furnace). Heating rate was constrained to $\frac{400}{Internal\;diameter\;(mm)},$ which for the 75 mm internal diameter work tube used produced a maximum heating/cooling rate of 5 °C·min^−1^ to minimise the risk of thermal shock. Sample bore was limited by the work tube internal diameter of 90 mm, especially as the sample-to-tube contact should be limited to minimise surface contact and subsequent thermal conduction. Although an isothermal load was required to provide representative in situ validation testing, the sample was inserted into a cold furnace to minimise the thermal stress on both the work tube and sample.

### 2.3. Metallographic Preparation and Imaging

Samples were mounted transversely in electrically conductive thermosetting resin (Bakelite). It was anticipated that oxidised samples may experience embrittlement, delamination, and spallation due to thermal stresses evolved during resin thermosetting. Consequently, the oxidised sample was therefore mounted in cold-setting resin before mounting in Bakelite to reinforce the oxide integrity during subsequent mechanical metallographic preparation. The fully mounted sample was ground to 1200 grit using silicon carbide (SiC) grinding paper and then polished using 50 nm colloidal silica solution, which was sufficient to reveal the substrate and scale microstructure without the need for chemical etching. Imaging was performed using optical light microscopy on Zeiss Primotech (Jena, Germany) and field-emission gun scanning electron microscopy (FEGSEM) where the optimal settings were an accelerating voltage of 12 kV and a probe current of 8 nA.

## 3. Results and Discussion

### 3.1. Oxide Microstructural Characterisation

**Figure 6:**
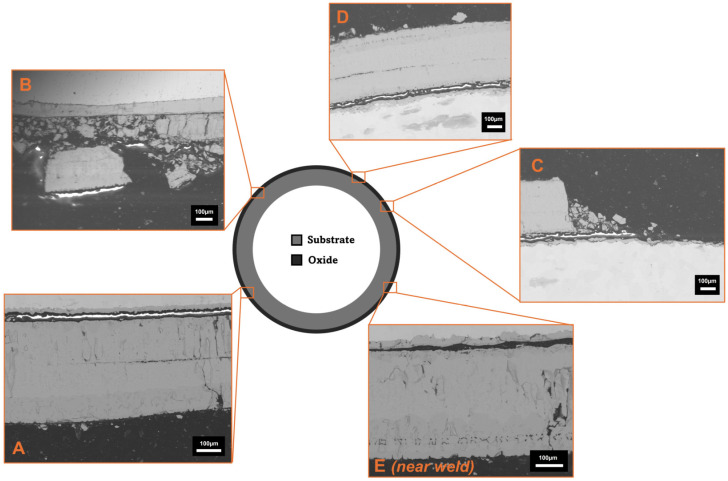
The schematic Schematic of positions (**A**–**E**) around the circumference of the outer surface of a 15 nominal bore (n.b.) tube where mean measurements of oxide thickness were made using Field Emission Gun Scanning Electron Microscopy (FEGSEM) after normalisation at 1000 °C for 15 min in a thermogravimetric analysis (TGA) furnace. Location E is near the tube weld seam.

**Figure 7:**
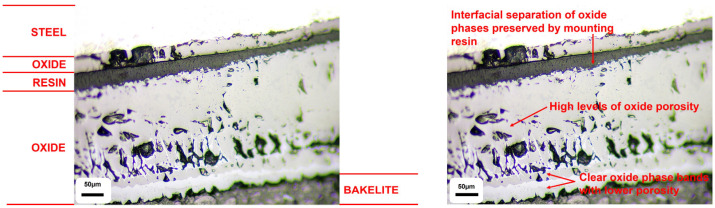
The optical microscopy inspection of oxide formed on the outer edge of 15 nominal bore (n.b.) conveyance tube sample oxidised in a thermogravimetric furnace at 1000 °C for 15 min. The left image highlights the phase regions whilst the right image highlights particular features related to oxide morphology and spallation risk.

**Figure 8:**
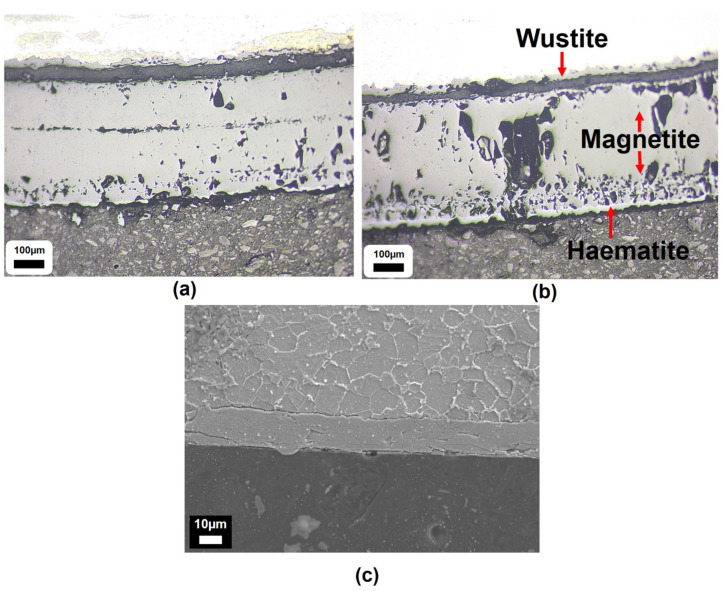
The light microscopy images of the outer edge of a 15 nb sample heated for 3 hrs in the TGA furnace, revealing (**a**) a thick, porous oxide of 401.0 ± 4.3 µm with (**b**) clear phases (wustite, magnetite, haematite) in the ratio and thickness range observed in Wilkstrom et al.’s work [57]. Equivalent industrial samples viewed via FEGSEM reveal a (**c**) much thinner, compact, homogeneous oxide of 15.8 ± 0.7 µm. In this case, the wustite, magnetite, and haematite phases (present at T > 833 K) are denoted as 3, 1, and 2, respectively, to match those of Wilkstrom et al. [57].

The qualitative results are presented below (see Figure 6 and Figure 7). Figure 8 highlights the thicker, porous, multi-phase oxide present on the experimentally grown sample compared to both the industrial and literature samples (see Figure 8). Although the thicker nature of the oxide grown in the TGA experiments, compared to industrial samples, made it easier to measure oxide thickness using optical light microscopy, FEGSEM was also used to more accurately evaluate the mean oxide thickness based on measurements taken at multiple locations around the tube circumference, as well as assessing any microstructural damage to the oxide. The average oxide thickness measured was 401.0 ± 4.3 µm, based on measurements taken at locations A and D, which displayed an intact, adhered oxide. This value lies within the uncertainty bounds of the less accurate optical light microscopy average oxide thickness measurement of 382.6 ± 63.5 µm.

In the isothermal soak time of 15 min, 223.5 µm of oxide is formed according to the model, which falls short of the lower limit, 396.7 µm, of the mean oxide thickness measured using optical microscopy. Although the model underpredicts the oxide thickness gain, it neglects the total time that the sample spends at a temperature where parabolic oxidation starts (2 hrs 15 min at 700 °C [54]). For the same conditions, the model predicts an oxide thickness of 670.6 µm, but the model is isothermal and therefore predicts this thickness for a sample soaked at 1000 °C, which is an overestimate. However, Kendall et al. [7] cited that their model is not equipped to include the transient regions of the TGA experiments and conveyance tube-manufacturing conditions, attributed to heating and cooling, i.e., non-isothermal conditions. A transient model yields steep thermal gradients and the temperature dependence of so many parameters in both the thermodynamic and kinetic databases, described by Kendall et al. [55], introduces significantly greater computational expense and reduces numerical stability. For example, diffusion coefficient, which contributes significantly to Kendall et al.’s model and databases, has an proportional relationship with temperature [55] and therefore needs to be recalculated for each temperature node within the transient region of the model. Changes in diffusion coefficient also affect oxide kinetics [56] and therefore oxide gain. Although Chen et al. noted that the exothermic nature of oxidation creates the potential for an overtemperature effect [54], which the model does not account for, this is most often in the case of small samples and limited equipment capability to conduct the heat away from the sample. The furnace work tube has an internal diameter of 90 mm; therefore, for a 15 n.b. sample with an OD of 26.9 mm, there should be sufficient clearance of over 30 mm on either side of the sample to allow heat to be conducted away. The oxide thickness gain observed in the sample lies almost equidistant between the under- and overprediction scenarios tested for the model. The mean of the values predicted is 447.1 µm (compared to 401.0 ± 4.3 µm measured on the TGA sample).

Arguably, the most significant factor is the surface condition of the sample pre-oxidation. Laboratory-based oxidation studies tend to prepare samples to a minimal level of surface roughness, using either 1200 or 2400 grit size grinding paper [58,59,60,61]. However, increased surface roughness can contribute to thicker scale, particularly in reference to its effect on external gas flow dynamics [34] and the stress state of the substrate and oxide where surface asperities promote imperfect adhesion and provide greater opportunity for oxygen penetration into unoxidised surfaces [62,63]. Humidity can have a similar, albeit less significant, effect on oxide kinetics and adhesion via the exposure of unoxidised surfaces. It has been shown that the adherence of scale to steel is improved and parabolic oxidation rate is increased significantly even when moving from dry to ambient air (1–2% water vapour) [54]. Scale in dry air was far more likely to separate from the substrate since water vapour can increase scale plasticity and creep [64,65].

**Figure 9:**
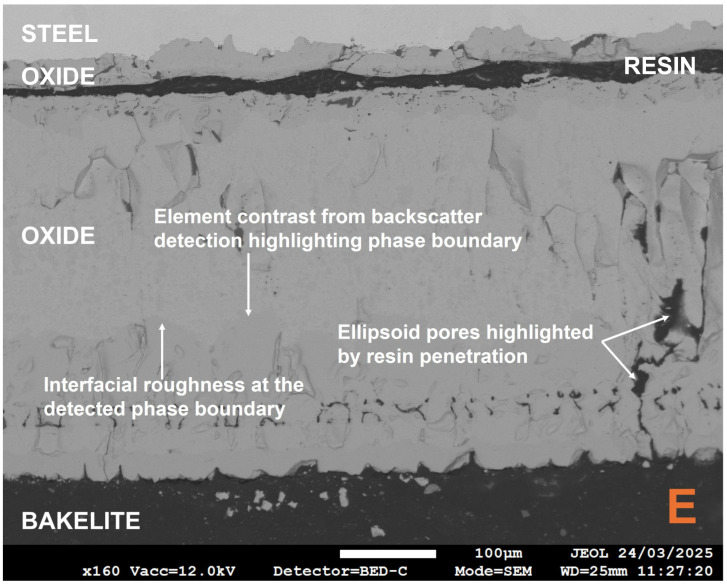
Annotated field emission gun electron micrograph, obtained via the backscatter electron detector, from location E showing the evidence of porosity, phase boundaries in the oxide, and interfacial roughness at some phase boundaries.

Locations B, C, and E were excluded from average oxide thickness calculations due to the extensive damage to the oxide found at that location and its surrounding area, with approximately 75% in a spalled state. The use of the backscatter electron detector (BED) within FEGSEM allows oxide phases to be detected based on atomic weight differences. The heavier iron element, with its larger nucleus, induces greater electron scatter, emitting more backscattered electrons and generating a brighter image [66]. For example, magnetite, Fe_3_O_4_, has a higher Fe:O ratio, i.e., a greater contribution of the heavier Fe atoms, and appears brighter than haematite, Fe_2_O_3_, both of which are typically adjacent phases in the multi-phase scales observed on low-carbon steel during conveyance tube manufacturing [8,21,22,67,68]. The different oxide phases observed showed varying porosity levels and interfacial boundaries (see Figure 9). Crucially, the oxide phases closest to the oxide–air boundary displayed evidence of delamination and the lowest interfacial strength and cohesion, whereas the innermost phase, 81.3 ± 8.8 µm, remained adhered to the steel, suggesting high metal–oxide interfacial strength and adhesion. The latter state was similar to that seen in an as-received industrial sample that had a compact, homogeneous oxide of 15.8 ± 0.7 µm, although the former has greater oxide thickness gain due to the lack of mechanical effects present during TGA testing, which can affect oxide kinetics [69].

**Figure 10:**
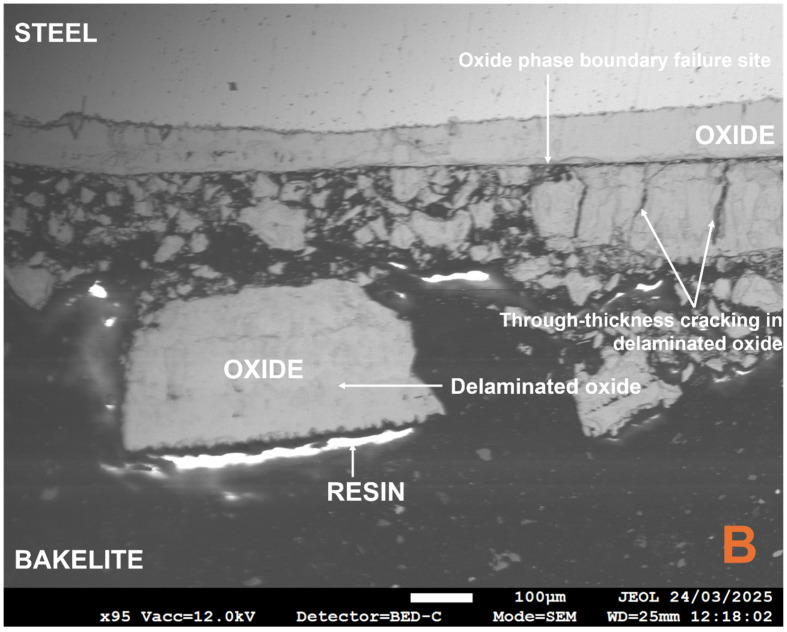
Annotated field emission gun electron micrograph, obtained via the backscatter electron detector, from location B showing the evidence of through-thickness cracking and interfacial failure.

Notably, the damage at location B (see Figure 10) highlights how the higher porosity, thicker, outer oxide layers delaminate along an oxide phase boundary, indicating lower interfacial strength and cohesion, whilst the most compact, thinner, innermost layers remain adhered to the steel, suggesting high metal–oxide interfacial strength and adhesion. It is argued that, in industry, this phenomenon could be attributed to the descaling process performed at the end of the tube-manufacturing routine, i.e., there is no opportunity for secondary scale formation, explaining the much thinner, more compact scale observed on industrial samples. Metallographic preparation appears to replicate this effect as it is analogous to the combination of thermal (hot mounting-induced), depositional compressive (oxidation), and impact (abrasive particles and lubricant) stress applied during descaling [65]. Hydraulic descaling is associated with a significant reduction in substrate grain size [70]. Smaller grains are associated with a greater number of grain boundaries, and at higher temperatures, grain boundary diffusion, $D_{gb},$ is favoured as a high diffusivity pathway, especially during the oxidation of iron [71], compared to its surface equivalent since the activation energy, Q, of grain boundary diffusion in most metals is 0.4–0.6 times the bulk diffusion equivalent, $D_{b}$, making grain boundary diffusion up to six orders of magnitude faster [72,73]. A simple mathematical analysis of Equation (10), the Arrhenius-type expression for diffusivity, demonstrates how the change in activation energy mentioned above translates to a scaling factor equivalent to the root associated with the change. For example, where grain boundary diffusion activation energy is half its bulk equivalent, the grain boundary diffusion coefficient is equivalent to the square root of the bulk equivalent.

(10) $$D=D_{0}e^{\left(-\frac{Q}{RT}\right)}$$

Hence, for values of bulk diffusion coefficient of below one, the most common scenario in diffusion analysis since $D_{b}\ll 1$, the grain boundary coefficient diffusivity is larger. Kendall et al. accounted for grain boundary diffusion in their model via a modified diffusion coefficient [7]. However, it should be noted that the thicker oxide formed during oxidation accelerated by grain boundary diffusion is eventually inhibited by the passive external layer of oxide formed that acts as a barrier to inward oxygen diffusion [74]. As previously discussed, changes in oxygen diffusion affect phase morphology, so scale growth accelerated by a greater degree of grain boundary diffusion is more likely to be dominated by wustite, where limited exposure to air is quickly achieved by the passive external oxide layer described [28].

However, the persistent evidence of scale present on the industrial samples, which have been exposed to a far greater level of mechanical damage, highlights the scale of the issue. It is clear that the varying surface condition on conveyance tubes is associated with inconsistent oxide spalling, arising from the oversimplification of the scale to a single body that can also be mechanically removed as a single body. In reality, the multi-phase nature of the scale is more accurately represented as multiple bodies whose adhesive and cohesive properties vary in the same way as their intrinsic mechanical properties [75]. Several authors have alluded to the importance of the interaction of phase transformations and descaling processes that were limited to planar samples, with the exception of Jiang et al. [27], and focused on other aspects of oxidation such as mechanical response [76], heat transfer behaviour [27,67], and microstructural characterisation [77].

However, since the model is limited to a single moving phase, and the biggest challenge arguably arises from the adhered inner layer that is resistant to hydraulic descaling, further uses of the model with respect to stress analysis will be limited to a compact layer of homogeneous oxide. It is still valid to assume that this homogeneous phase will be an oxide as it is typically found closest to the metal–oxide reaction interface as it has the lowest oxygen content of the three phases typically detected in an oxide formed on low-carbon steel [54]. The oxide is therefore assumed to have already been exposed to descaling-related stresses, as described by Basabe and Szpunar [65], during stress analysis, rather than adding further complexification. Furthermore, silicon (Si) strongly influences carbon steel oxidation behaviour as an element capable of segregating the scale–metal interface at high temperatures to form diffusion-inhibiting fayalite (Fe_2_SiO_4_) [57,59,78,79]. Although steel–scale adhesion is lower on Si steels than on pure iron, the silica and/or fayalite (Fe_2_SiO_4_) to substrate adhesion is stronger and introduces descaling challenges [80]. Wilkstrom et al. [57] described how, at high reheat temperatures, the low thermal gradient evolved between the slab and atmosphere indicating that a fast exothermic fayalite reaction initiates, and the development of significant latent heat and poor heat transfer conditions melts the fayalite and Fe-O systems, causing enhanced diffusion that generates further heat. The application of this observation was highlighted in Wassilkowska et al.’s work as the potential to improve corrosion resistance by easily mechanically removing the top oxide layer to expose the silicon-rich layer [6]. Whilst this well-adhered layer is advantageous in experimental contexts and when attempting to preserve the protective passivating oxide on the inner surface of tubes (which is beyond the scope of this work), it also promotes the incomplete descaling outlines as a key issue for the external tube surface during conveyance tube manufacturing. Krzyzanowski et al. cited the quantification of interfacial separation stress as a key area for further work, where silicon content is clearly important [78]. As such, incorporating silicon into models of oxidation kinetics is also identified within this manuscript as an avenue for future work.

**Figure 11:**
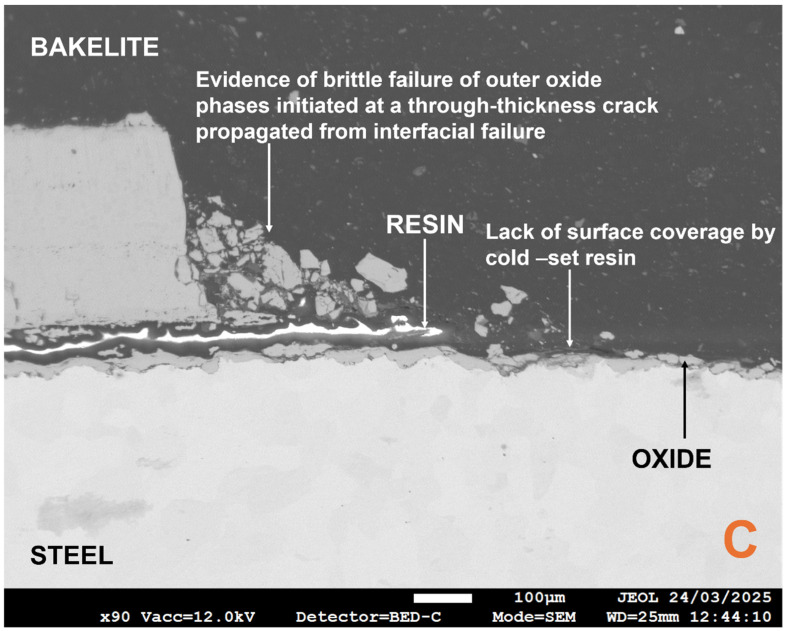
Annotated field emission gun electron micrograph, obtained via the backscatter electron detector, from location C showing the evidence of interfacial failure-initiated through-thickness cracking in the absence of sufficient cold-set resin coverage. The thinner compact layer of homogeneous oxide remains intact and adhered.

In the case of interfacial oxide phase failure, the use of cold-setting resin to seal the oxide in its post-TGA test state prior to metallographic preparation is advantageous in distinguishing between thermomechanical and sample preparation-related damage. For example, in Figure 9 and Figure 10, the resin has preserved the interfacial separation of the thinner inner and thicker outer oxide phases. The gap present can then be locked in place by the hot mount resin. This is further reinforced by Figure 11 where there was a gap in the coverage of the oxide by the cold-setting resin. The abrupt end in the thick oxide layer followed by a very thin layer highlights the importance of selecting the appropriate mounting technique, as well as supporting conclusions about the effect of descaling on oxide thickness and microstructural topography in laboratory vs. industrial settings.

The use of the backscatter electron detector (BED) again highlights how the spalled scale is an oxide phase duplex and emphasises why the scale observed on industrial samples, in contrast with traditional scale growth kinetics [24], is homogeneous. Furthermore, the literature highlights how the outermost oxide phase has the highest tensile strength value [75], but the dominance of interfacial failure highlights how the adhesion and cohesion of the oxide (and steel) is more important for oxide damage during tube manufacture than intrinsic mechanical properties. Furthermore, there appears to be much higher levels of interfacial roughness for the two outermost oxides, which are ultimately spalled. This has also been shown to negatively affect both kinetic and adhesion behaviour [62,75,81]. However, it should be noted that failure mode is temperature-dependent, and mechanical properties may be more important for oxides formed at lower temperatures [78]. The challenge of porosity has already been discussed by other authors [28,69,82], and was addressed by Kendall et al. [7], in particular how pores become larger and non-uniformly distributed as the oxide grows on a curved geometry and the outermost oxide moves further from the metal–oxide reaction interface, thus making the model less accurate [69]. The pores in Figure 9 are highlighted by infiltration with the cold-set resin and agree with the trend proposed by Entchev et al. [69]. Entchev et al. [69] highlighted how porosity becomes less uniform and pores become larger as the oxide thickens and preceding oxide layers expand. Porosity can become problematic when pores expand to restore the equilibrium concentration of vacancies, which optimises configurational entropy and defect formation enthalpy. However, it must be noted that Yamaguchi and Someno [83] identified that oxygen diffusion in wustite is a function of oxygen potential so the dominant diffusion mechanism must be via interstitial cations. Furthermore, much of the microstructural and microtextural properties of the laboratory-grown scale, e.g., ridges, roughness, through-thickness cracking, interfacial cracking, and severe spalling, are typical of laboratory scale formed in air, i.e., heating atmosphere has been shown to be important [28,76,84]. Wang et al. highlighted that electrochemically grown oxides, i.e., oxides with limited exposure to air, are far more likely to be compact, homogeneous, uniform, and intact [28]. Hence, the oxide of the same description found in industrial samples can only have grown with limited access to air caused by the existing layers of oxide above its outer surface. This further supports the idea that descaling causes delamination as opposed to any spalling during the oxidation reaction itself, since then the outer layers of oxide on the industrial sample would begin to show characteristics of air-based oxidation. Previous work has highlighted that poor scale adhesion is ultimately a combination of kinetic and mechanical phenomena [85], and the kinetic phenomena that propagate circular pore formation are deformed to ellipsoids (observed in Figure 9) under compressive thermal and growth stresses [28,86]. However, it could be argued that the excellent mass gain agreement between the model and TGA sample is skewed by the presence of significant porosity in the majority of the oxide formed on the TGA sample, which would decrease the oxide density [44]. For example, estimations of porosity in wustite vary between 10 and 44% [64,83,87,88]; therefore, in the worst case scenario, the volume of oxide, $V_{ox}$, and subsequently calculated density could be almost half of their expected values (see Equation (11)).

(11) $$\rho_{ox}=\frac{m_{ox}}{V_{ox}}$$

Therefore, the mass change is reduced compared to when the oxide was fully compact. Introduction of porosity into oxide kinetic modelling is another avenue for future work.

### 3.2. Quantitative Oxide Gain Analysis

Although without continuous data the experimental and industrial samples are not comparable due to the difference in soak time, the model demonstrates its advantage in flexibility and continuous data prediction and suggests a mass gain of 1.52 g for the same thermal cycle as experienced by the experimental sample (which gained 2.00 g based on pre- and post-test weight measurement). Model underprediction is likely multi-factorial owing to the absence of an adequate technique to include porosity in the model, except via diffusion coefficient modification, as well the effect of stress in both the oxide and substrate, the analysis of which is beyond the scope of this work. Furthermore, where the mass balance is not integrated into the TGA apparatus, there is always a significant risk of material loss during sample transfer, especially given the brittle nature of the oxide evolved on conveyance tube steel grades [26,27,28,29].

**Figure 12:**
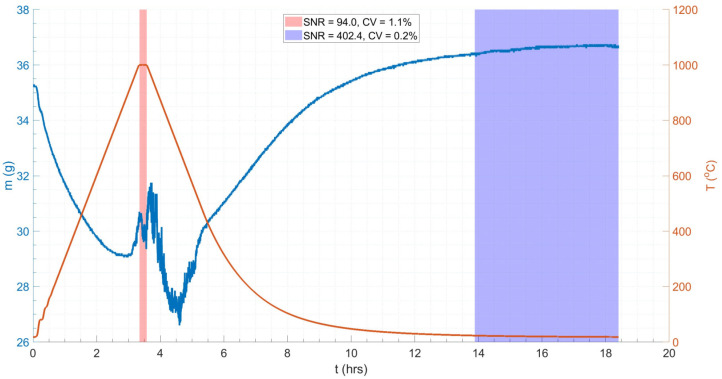
The plot of mass change (**left**) and temperature (**right**) against time for a tube normalisation heat treatment replica thermal cycle on a 15 n.b. tube sample. Annotated to include evaluation of signal-to-noise ratio (SNR) and coefficient of variance (CV) in the isothermal (**left**) and non-isothermal (**right**) data regions. The higher SNR and lower CV in the non-isothermal data region indicate higher quality data with less noise.

Figure 12 shows the mass and temperature profiles of the 15 nb sample subjected to the same thermal cycle as the ceramic dummy. The mass of the sample post-test, measured again using the Fisher Scientific mass balance, was 35.8579 g, indicating an oxide mass gain of 1.6583 g. The sample exhibited a 19% mass loss during the heating and cooling periods of the thermal cycle, and significant signal-to-noise ratio (SNR) and coefficient of variance (CV) of 94.0 and 1.1%, respectively (based on the mean, μ, and standard deviation, σ, values) during the isothermal soak period (red region in Figure 12), compared to the less noisy region post-cooling (blue region in Figure 12), which had an SNR and a CV of 402.4 and 0.2%, respectively (see Equation (10)) [51]. A higher SNR and lower CV typically indicate better quality data. The latter (post-cooling) region acts as a data quality baseline, i.e., noise is minimised to only a statistical level [89]; hence, the four-fold reduction in SNR in the isothermal region is reflective of the reduction in data quality associated with VIV. Disregarding the subsequent mass losses and gains over the thermal cycle, the net mass change was −0.3 g (see Figure 12).

**Figure 13:**
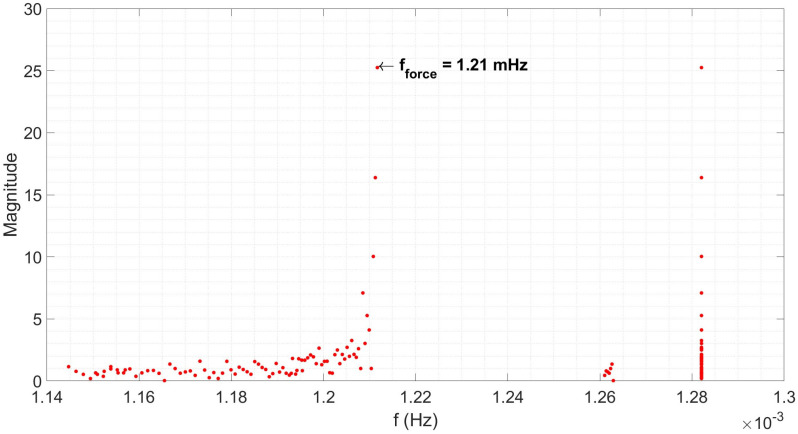
The frequency spectrum for the isothermal period of a 1000 °C normalisation heat treatment cycle on a 15 n.b. steel conveyance tube.

The results are shown in Figure 13 and clearly show 1.21 mHz as the pendulum resonance frequency, assuming the peak observed at the lowest frequency is the first harmonic.

**Figure 14:**
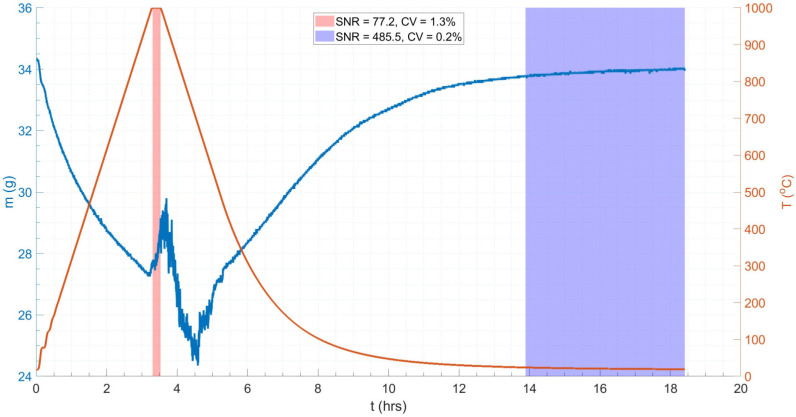
The mass and temperature profiles for a representative normalisation cycle using a refractory (Al_2_O_3_) ‘dummy’ sample. Annotated to include evaluation of signal-to-noise ratio (SNR) and coefficient of variance (CV) in the isothermal (**left**) and non-isothermal (**right**) data regions. The higher SNR and lower CV in the non-isothermal data region indicate higher quality data with less noise.

Figure 14 shows the results of the dummy test. A net mass change of −0.3 g was observed post-test once the minor oxidation of the nichrome wire had been accounted for. Again, there is a large (six-fold) reduction in SNR during the isothermal test region (red in Figure 14) compared to the stable region (blue in Figure 14), which matches that observed in the steel sample, indicating that data quality is affected by VIV.

**Figure 15:**
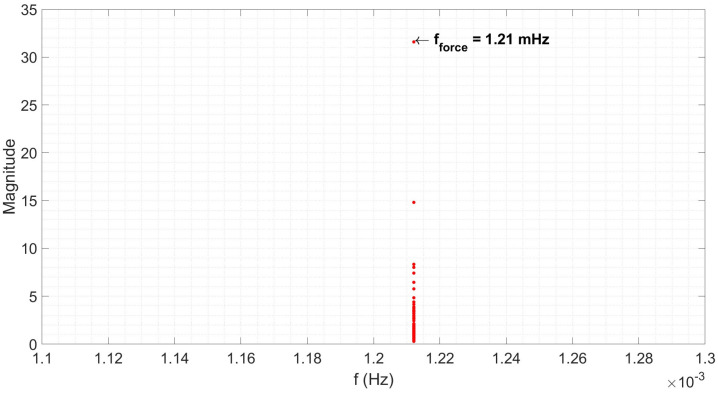
The frequency spectrum for the isothermal period during a 1000 °C normalisation heat treatment cycle on an alumina tube.

**Figure 16:**
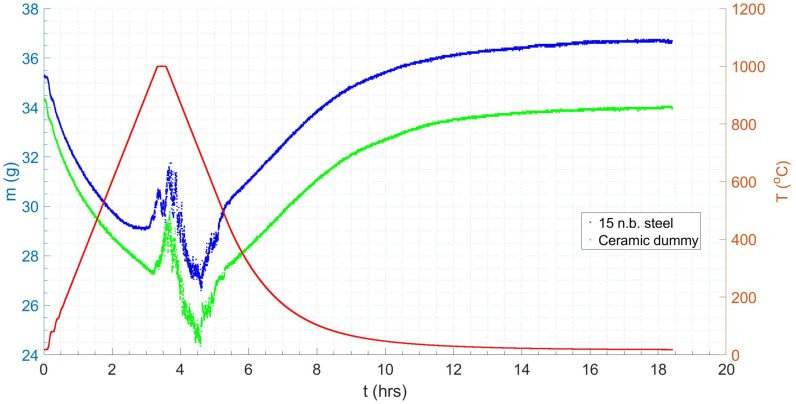
The combined mass–temperature plots for the steel (*blue*) and ceramic (*green*) samples following a representative normalisation thermal cycle. The temperature profile is denoted by the red line.

**Figure 17:**
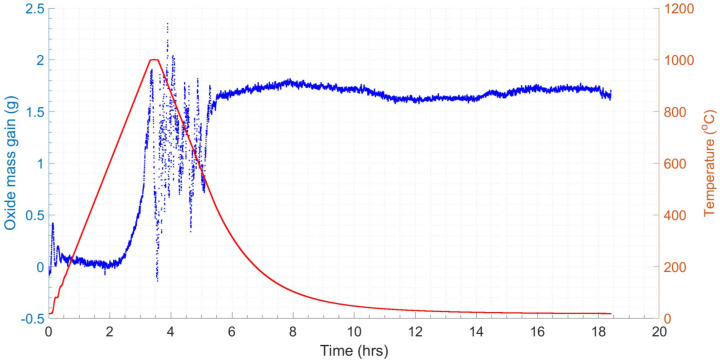
Combined mass (blue)–temperature (red) plot for oxide mass gain on 15 nominal bore (n.b.) sample.

**Figure 18:**
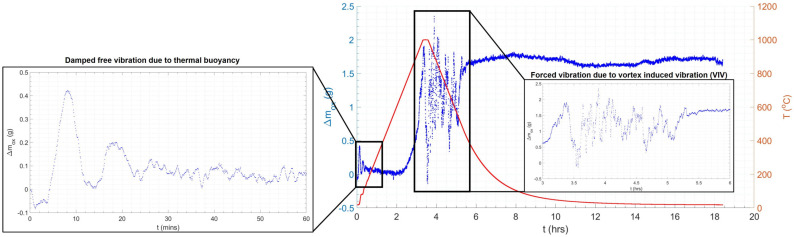
Combined mass (blue)–temperature (red) plot for oxide mass gain on the 15 nominal bore (n.b.) sample with enlarged regions exhibiting damped oscillation during the first hour of mass measurement during the heating period (free) and at the onset of the isothermal soak and initial cooling period (forced).

**Figure 19:**
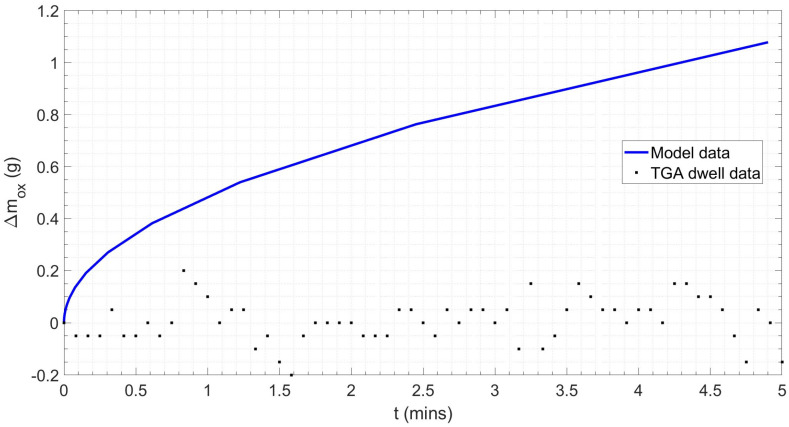
Oxide mass gain observed during model simulation and TGA dwell data for a 15 n.b. tube during a normalisation heat treatment cycle (5 min soak) at 1000 °C.

Figure 15 shows the results of the FFT applied to the ceramic sample results, resulting in a 2 d.p. agreement between the forcing frequency for the steel and ceramic samples, indicating the consistency of the vibration effect. However, only a single forcing frequency was observed for the ceramic sample, whilst the steel sample had evidence of a secondary harmonic, which was potentially produced by the vibration of the steel–scale interface [90]. The profiles for the dummy and real tests were overlaid (see Figure 16). The difference in mass, given the similar profiles, was taken to be the oxide mass gain, once the initial difference in mass between the two samples was subtracted. A plot of oxide mass gain against time is shown in Figure 17. There are discretised data where mass change, at the sampling rate of 0.2 Hz, is less than the mass balance resolution so a moving average filter with a window of 9 was applied. There is clear evidence of free damped oscillator motion, described in Section 2, in the first hour of mass measurement during the heating period and at the onset of the isothermal soak and initial cooling period; although in the latter cases, the damped oscillator motion appears forced, before mass achieves a stable value (see Figure 18) [91]. Figure 19 compares the results in Figure 17 with those predicted by the model.

**Figure 20:**
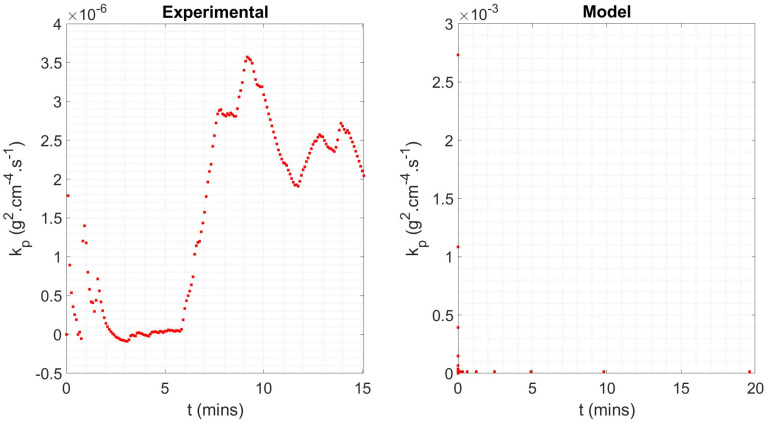
Instantaneous mass-based parabolic rate coefficient plotted against time for the isothermal period of thermogravimetric (‘experimental’) testing (**left**) and model (**right**) data, which lasts approximately 15 min.

However, once the challenges associated with sample vibration have been mitigated during the experiment and/or post-processing, overall, there is very good agreement between the model and experimental datasets. For the same sample geometry, OD = 26.9 mm and $r_{wall}$ = 3.2 mm, and isothermal conditions, 1000 °C for 5 min, the oxide mass gain predicted by the model was 1.52 g, whilst the sample in the experiment gained 1.60 g of oxide, a difference of only 5%. The mass-based parabolic rate coefficient, $k_{p}$, for the model and experiment was 1.6752 × 10^−5^ g^2^·cm^−4^·s^−1^ and 2.0435 × 10^−6^ g^2^·cm^−4^·s^−1^, with the experiment exhibiting oxidation at only 12% of the rate of the model. However, the difference is partially due to the idealised parabolic equation used to define the model, and in greater part due to the severe fluctuation in mass gain observed during the isothermal period of TGA testing. Figure 20 shows how $k_{p}$ maintains a stable value of 1.4133 × 10^−5^ g^2^·cm^−4^·s^−1^ as oxidation proceeds during model-based normalisation, after a short initial period of rapid decrease due to the parabolic nature of the oxide growth. However, $k_{p}$ fluctuates across a range of 3.6596 × 10^−6^ g^2^·cm^−4^·s^−1^ during the isothermal period of the TGA (‘experimental’) test. The mean of the instantaneous $k_{p}$ value reflects this at 5.2234 × 10^−5^ g^2^·cm^−4^·s^−1^ and 1.5209 × 10^−6^ g^2^·cm^−4^·s^−1^ for the experiment and model, respectively. Hence, the discrepancy is therefore a product of the experimental challenges previously discussed rather than issues with the model.

**Figure 21:**
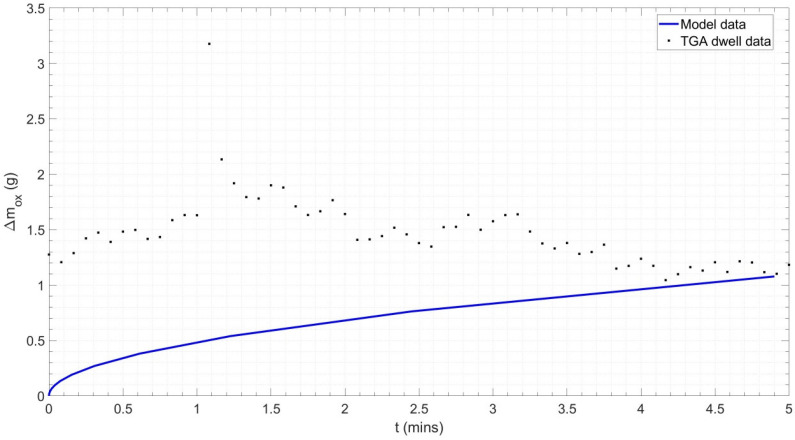
Oxide mass gain observed during model simulation and TGA dwell data for a 15 n.b. tube during a normalisation heat treatment cycle (5 min soak) at 1000 °C. The TGA dwell data were processed with Fourier transform operations to suppress resonance caused by vortex-induced vibration.

**Figure 22:**
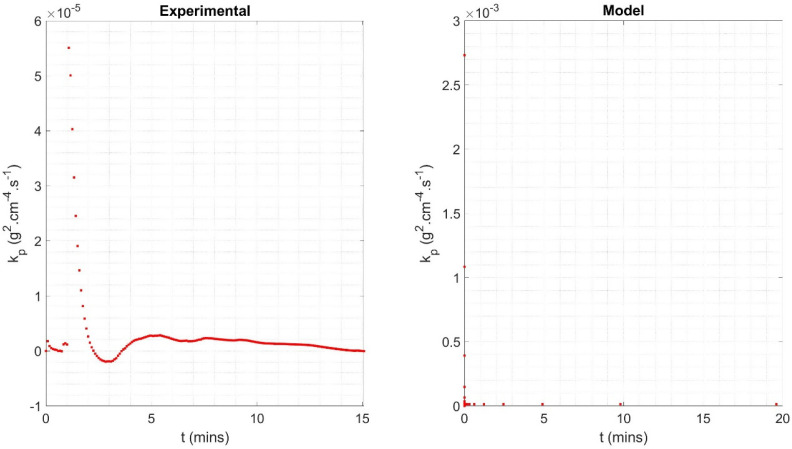
Instantaneous mass-based parabolic rate coefficient plotted against time for the isothermal period of thermogravimetric (‘experimental’) testing (**left**) and model (**right**) data, which lasts approximately 15 min. The experimental data were processed with Fourier transform operations to suppress resonance caused by vortex-induced vibration.

The resonant frequency associated with VIV, $f_{force}=$ 1.12 mHz, was used to improve the agreement between the experimental and computational oxide mass gain by its pointwise subtraction from the frequency-series experimental oxide mass gain dataset. The resulting resonance-suppressed data were returned to their time-series form via an inverse Fourier transform (see Equation (7)) and processed in the same way as the original data. Figure 21 shows the improved agreement between computational and experimental data, with an improving agreement with time. This is supported by the evidence of a stable instantaneous value of mass-based parabolic rate coefficient of 2.5 × 10^−6^ g^2^·cm^−4^·s^−1^ after normalisation (t = 5 min) in Figure 22.

As the most discrepancy is between the computational and experimental data in the first two minutes of isothermal heating, this indicates some residual instability from the thermally induced free vibration observed in the heating period. This project is most concerned with the isothermal period associated with normalisation and therefore addresses mitigation strategies for VIV. However, separate considerations for the specific physical features of thermal buoyancy-induced free vibration could be addressed in further work. Additionally, oxide mass gain data from the computational and experimental simulations of longer industrial heat treatments, e.g., hot mill reheating, could be used to investigate whether the improved agreement is sustained as oxidation proceeds further at a constant soak temperature.

## 4. Conclusions

An industrially supplied sample of a low-carbon steel conveyance tube, in its as-welded state, was subjected to thermogravimetric analysis (TGA) during the laboratory furnace-based replication of a typical tube normalisation thermal cycle. Thermally induced gas flow dynamic phenomena, and their induction of thermal buoyancy, forced vibration, and pendulum phenomena, were proposed as the greatest sources of noise and mass loss during the TGA test. Mitigation steps, e.g., shorter suspension wire and limited sample tilt, were accompanied by a separate identical test of a refractory alumina ‘dummy’ sample to isolate and quantify the pendulum effect. The pendulum effect was subtracted from the steel sample TGA data to obtain continuous oxide mass gain data. After TGA testing, the sample was compared with a tube of the same dimensions which was normalised in industry, as well as the results of an existing 2D oxidation model. The computational approach, though a simplification of complex real conditions, offers an alternative approach without the practical issues described.

- Post-test inspection of the TGA data revealed a 5% discrepancy with the mass gain predicted by the model once damped oscillator motion as the buoyancy and vortex-induced forced vibrations effects were accounted for.
- The multi-phase oxide formed during TGA testing (1000 °C isothermal soak for 15 min with 5 K.min^−1^ heating and cooling ramps) was porous with a mean thickness of 401.0 ± 4.3 µm. The oxide phases closest to the oxide–air boundary displayed evidence of delamination and the lowest interfacial strength and cohesion, whereas the innermost phase remained adhered to the steel, suggesting high metal–oxide interfacial strength and adhesion. The latter state was similar to that seen in an as-received industrial sample that had a compact, homogeneous oxide.
- Although Kendall et al.’s model [7] underpredicts the oxide thickness gain, it does not account for the time spent at a temperature where oxidation is significant (>700 °C). For the same time spent at 1000 °C, the model predicts 670.6 µm, which is an overprediction given that the sample experiences a temperature change of 300 °C in the same time.
- There are also general factors that can affect oxide kinetics, to varying degrees, which are not accounted for in the model and/or experiments described, e.g., surface roughness, humidity, and porosity.
- The much thinner, homogenous oxide observed on the industrial sample was attributed to the lack of replication of mechanical operations, especially hydraulic descaling associated with conveyance tube manufacturing, during experimental and computational investigations.
- Overall, successful experimental and industrial validation revealed a scope for the computational model to be refined in future work to better reflect specific complexities associated with manufacturing processes such as conveyance tube manufacturing, e.g., transient heating, porosity, and mechanical effects during manufacturing such as hydraulic descaling.

## Figures and Tables

**Table 1:** Alloy element composition limits specified by BS EN 10217-2 (welded steel tubes for pressure purposes—technical delivery conditions [Part 2: electric welded non-alloy and alloy steel tubes with specified elevated temperature properties]) for steel grade P235GH [35].

P235GH	C	Si	Mn	P	S	Cr	Ni	Al	Cu	Nb
**% wt**	≤0.16	0.35	≤1.20	0.025	0.020	0.30	0.30	0.020	0.30	0.010

**Figure A1:**
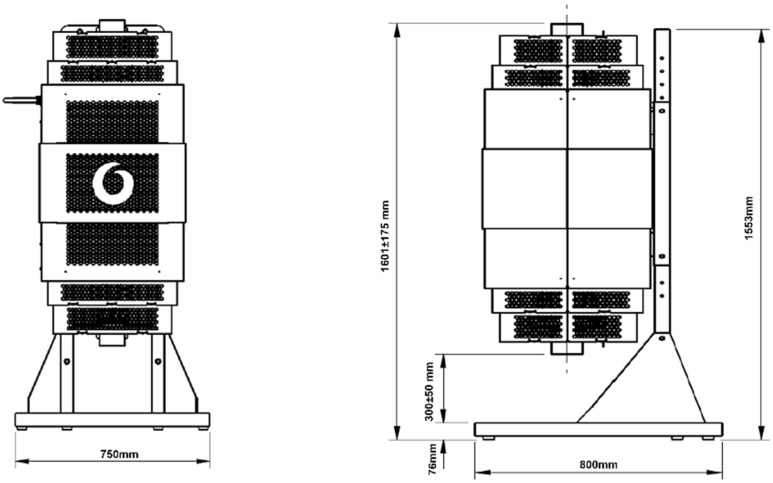
Technical drawing (taken from model TF1 16/100/450 furnace manual) of vertically mounted Carbolite Gero Ltd. high-temperature TGA tube furnace (model TF1 16/100/450) (adapted from source).

**Figure A2:**
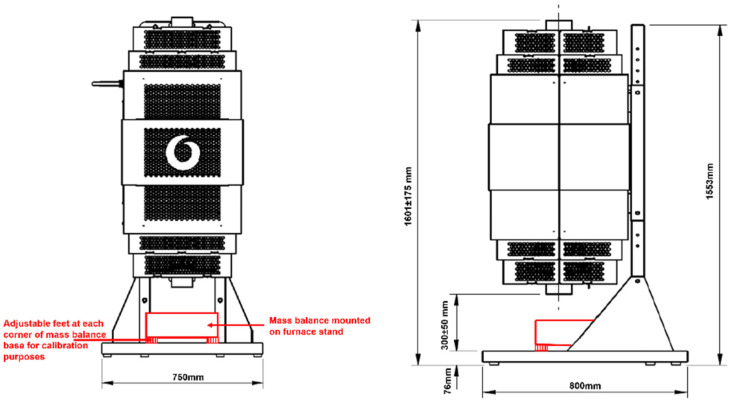
Technical drawing (taken from model TF1 16/100/450 furnace manual) showing the incorporation of a mass balance into vertically mounted Carbolite Gero Ltd. high-temperature TGA tube furnace (model TF1 16/100/450).

**Figure A3:**
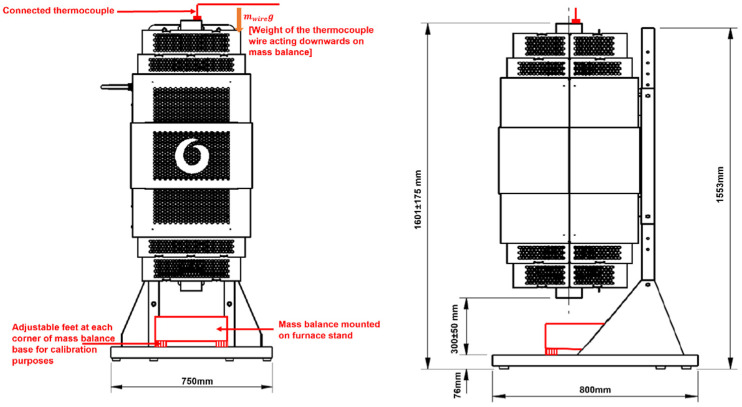
Technical drawing (taken from model TF1 16/100/450 furnace manual) showing the incorporation of a mass balance and thermocouple into vertically mounted Carbolite Gero Ltd. high-temperature TGA tube furnace (model TF1 16/100/450).

**Figure A4:**
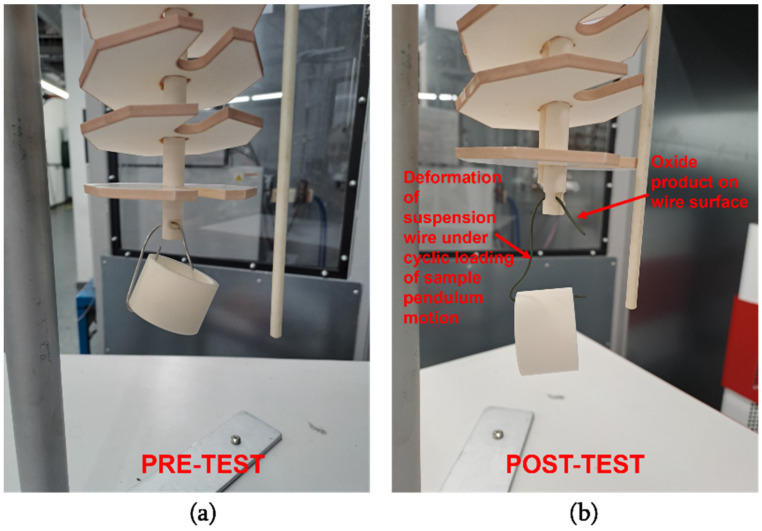
The comparison of ceramic sample (**a**) before and (**b**) after normalisation treatment. The nichrome suspension wire shows the evidence of oxidation and deformation under the cyclic loading of the sample pendulum motion.

## Data Availability

The datasets presented in this article are not readily available because this work is part of ongoing study and is currently commercially sensitive. Requests to access the datasets should be directed to M.K.

## Abbreviations

The following abbreviations are used in this manuscript:

BED	backscatter electron detector
CV	coefficient of variance
d.p.	decimal places
DFT	discrete Fourier transform
FEGSEM	field emission gun scanning electron microscopy
FFT	fast Fourier transform
HVAC	heating ventilation and air conditioning
n.b.	nominal bore
SNR	signal-to-noise ratio
TGA	thermogravimetric analysis
VIV	vortex-induced vibration

## Appendix A. Troubleshooting Experimental Issues for Curved Surfaces and Thermogravimetric Analysis (TGA)

### Appendix A.1

This testing faced several technical challenges; with the first being the ‘overload’ of the mass balance during sample loading with a sample weighing only 0.3% of the maximum 15 kg load possible for the mass balance model used. This invalid overload was attributed to incremental movement of the furnace tube due to persistent laboratory environmental vibration and thermal currents during previous temperature data-only collections (where the issue would not be detected). The movement resulted in the work tube no longer being isolated from the mass balance and its own mass caused an overload reading. The furnace tube was repositioned and the correct position was recorded by marking the adjustable (for calibration purposes) mass balance feet with a pen for future reference (see Figure A1 and Figure A2).

The pre-test mass balance reading with the sample installed and the thermocouple connected lacked stability due to the downwards movement of the thermocouple wire under its own weight (see Figure A3). Therefore; the first trial was run without the thermocouple connected and during post-processing; and the furnace temperature data were instead taken from the Eurotherm nanodac data acquisition unit. The choice of temperature data source will make a difference to measurement uncertainty as the spatial temperature variation in the furnace work tube means there may be a discrepancy between sensors in different locations; e.g.; sample thermocouple vs. furnace control thermocouple.

### Appendix A.2

Figure A4 shows an issue with cyclic loading and creep of the nichrome wire due to the free movement of the sample on the wire during pendulum motion under forced vibration. This caused the nichrome wire to open and put the sample at the risk of falling from the sample hook during test; or during transfer after testing. For brittle materials where drilling a through-thickness hole is not feasible; it is recommended that the wire is secured in place.
